# The Clinical Value of a Leucocyte Count in the Diagnosis of Septic Infections1Read before the January meeting of the Minnesota State Veterinary Medical Association.

**Published:** 1900-03

**Authors:** George Douglas Head

**Affiliations:** Instructor in Pathology in the University of Minnesota


### THE JOURNAL

OF

### COMPARATIVE MEDICINE AND VETERINARY ARCHIVES.

VOL. XXI. *MARCH, 1900.* No. 3.

### THE CLINICAL VALUE OF A LEUCOCYTE COUNT IN THE DIAGNOSIS OF SEPTIC INFECTIONS.[1]

BY GEORGE DOUGLAS HEAD, B.S., M.D.,
INSTRUCTOR IN PATHOLOGY IN THE UNIVERSITY OF MINNESOTA.

THIS paper was originally written to be read before a society of medical men who deal with septic infections in man. What is said, however, concerning the value of the leucocyte-count in detecting pus-formations in human beings can be applied by veterinarians to septic infections in the higher animals.

A limited observation seems to indicate that the number of leucocytes in the circulating blood of the higher animals, such as the dog, cow, horse, pig, rabbit, cat, etc., is nearly the same as that in man, and, further, that these leucocytes are increased or diminished in number in man and the higher animals by like causes.

White blood-counts in five healthy dogs showed the following: 9600, 9000, 6600, 7300, 8000, or an average of about 8000 leucocytes to the c.mm., a number practically the same as the normal number of leucocytes in the circulating blood of man. In twenty healthy rabbits counted by the writer the number of white cells in the circulating blood averaged about 8000, the same as in the dog and man. Although the writer has not made counts upon other animals, he feels certain that all the higher animals have approximately 8000 white corpuscles to the c.mm. in the circulating blood.

When we come to deal with septic infections in higher animals the writer has made counts in enough instances to make it altogether probable that in this class of pathological conditions the number of leucocytes in the circulating blood of the higher animals is increased the same as in man. Take dogs, for example. Septic infection seems to be followed by a well-marked leucocytosis.

[1] Read before the January meeting of the Minnesota State Veterinary Medical Association.

In one dog that had a normal count of 8000, and that subsequently developed a septic infection in the right foreleg with the formation of a focus of pus, the leucocyte-count was 20,600. In another dog that developed an abscess at the site of an operation the white-cell count was 36,300 ; his count previous to the abscess was 9200.

In rabbits, also, a septic infection seems to be followed by a pronounced leucocytosis. In a series of experiments which the writer undertook to determine the white blood-count in rabbits infected with rabies, Rabbit No. 63 up to two days of its death counted 7300 white cells to the c.mm., then for some unknown reason the count mounted up to 16,000, a very unusual observation, since rabies does not cause a leucocytosis in rabbits. Thinking I had erred in the count, I made a recount, with 20,600 as the result. Still being skeptical, I waited some hours, and recounted the rabbit's blood and found 15,000 white cells to the c.mm. I could not account for this leucocytosis upon any other supposition than that this rabbit harbored some complicating conditions, probably a septic infection. At post-mortem a small abscess was found at the seat of inoculation.

The writer feels certain that septic infections in the higher animals produce a leucocytosis similar to that in man, and that what may be said in this paper regarding the leucocyte-count in septic infections in man may be directly applied to the higher animals.

Leucocytes are present in the circulating blood of the human adult in the number of 7000 to 8000 to the c.mm. In children the number is somewhat higher. Infants of one year or less average about 12,000, while in older children the count is 8000 to 9000 corpuscles to the c.mm. Under certain conditions the number of white cells in the human blood may be increased beyond the normal limits. Such an increase may be very great, reaching 30,000 to 50,000 to the c.mm., or it may be only slight, 10,000 to 15,000 to the c.mm. Any increase in the number of leucocytes of the circulating blood is called leucocytosis, or, as some authors prefer, hyperleucocytosis.

What forces act to occasion this rise in the number of white cells in the blood has not been definitely determined. Leucocytosis has been theoretically explained by Virchow and Ehrlich, who consider it due to a stimulation of the blood-making organs, and consequent over-production of white cells.

The theory which, it seems to me, best explains leucocytosis is that advocated by von Linibeck, Jakob, and Goldschneider. These observers take the stand that there is no actual manufacture of new white cells in the production of leucocytosis, but that the bacterial toxins circulating in the blood act in a chemotactic way to attract into the blood-stream leucocytes which were before stationed in the lymph-spaces, spleen, and lymph-nodes, and that these leucocytes, added to those already in the blood, cause the higher white blood-count and the phenomena known as leucocytosis.

It does not matter, in the consideration of this subject, how we explain the phenomena theoretically. We know that it exists, and that under certain conditions the leucocytes of the circulating blood may be increased beyond the normal limits. Some of these conditions are physiological, such as the leucocytosis of pregnancy, of exercise, of digestion, and the like. Such an increase of white cells is of importance from a pathological point of view only that the clinician making white counts with the patient in such physiological states must bear in mind the leucocytosis thus produced. Other conditions causing an increase of white cells are pathological. Such are the leucocytosis of pneumonia, rheumatism, erysipelas, diphtheria, scarlet fever, secondary anæmia, and especially all forms of septic infections.

It is to this latter condition, namely, the presence of pus in the body and the consequent leucocytosis which it occasions that your attention is directed. The presence of pyæmic infection in the body is usually attended by a well-marked increase of white corpuscles in the circulating blood. This fact holds true, whether the infected point be a furuncle on the back of the neck, an abscess in the middle of the ear, or a purulent inflammation in a joint. Even if the process is more severe and involving more important structures, such as the vermiform appendix or the Fallopian tube, this same influence in the number of leucocytes in the circulating blood is always present if the inflammation has gone on to suppuration.

The height to which the white blood-corpuscle count may rise varies according to the severity of the inflammatory process, the location of the infection, and the duration of time which has elapsed since the pyæmic process first began. If the inflammation has continued for some time, say three or four weeks or more, and the pus has been well walled off from the surrounding structures, the leucocytosis will not be as large as if the process were in its first stages. For example: Some months ago the writer counted the leucocytes of a patient who had a swelling in the right abdomen for twelve years. The white blood-count was only 11,000 leucocytes to the c.mm—a count too low to make a positive diagnosis of an abscess. Yet, when subsequently the patient was operated upon, the swelling was found to be composed of pus. Had this patient been seen early in the history of the case the leucocyte-count would in all probability have reached 20,000 or 30,000 white cells to the c.mm., and a correct diagnosis of the existing conditions made. Here, then, is an exception to the general rule of a well-marked leucocytosis with pyæmic processes, namely, when the inflammation has been of long standing and the pus well walled off from the surrounding structures.

There is also another exception which must here be noted. In some instances where the septic infection is most severe and rapidly causes fatal results no leucocytosis will occur as a consequence of the infection. An example of such exceptions is the rapidly fatal result of septic peritonitis where the patient is attacked suddenly and is quickly overcome by the disease. In these cases no increase of white blood-corpuscles will be found in the circulating blood.

Aside from these two exceptions we may adduce the general law, "that in all cases of septic infection accompanied by the formation of pus a well-marked leucocytosis results." By a well-marked leucocytosis we mean a white blood-count of about 15,000 or more leucocytes to the c.mm., the normal count in a human adult being considered 7500 white cells to the c.mm.

The practical value of this knowledge to the physician and veterinarian, once it is secured, cannot fail to impress itself upon your minds. For example: Many times the question arises, Does or does not this swelling contain pus? In the experience of the medical man that swelling may be deep in the abdomen, where a rigid abdominal wall and an absence of characteristic symptoms would make a diagnosis almost if not quite impossible without an estimation of leucocytes. In the experience of the veterinarian that swelling may be of such a nature as to baffle the diagnostician. Under such conditions the white blood-count will give a clue to the character of the inflammation and assist the clinician to a correct diagnosis. Again, repeated white blood-counts of cases of suspected septic infection will give positive evidence as to whether the process is extending and involving new areas of tissue or whether it is well localized and walled off from the surrounding structure. If the white blood-count increases from one day to the next the inflammation is spreading. If the count is stationary and there is neither an increase nor a diminution in the number of white cells the process is not involving new areas of tissue. If the leucocyte count diminishes from day to day the infective process is receding, recovery by resolution can be anticipated, and no operative interference is needed. Again, in the determination of the question, is the swelling cystic, hemorrhagic, serous or purulent, the leucocyte count is of great value. Cystic, serous, or small hemorrhagic swellings cause slight, if any, leucocytosis, while a pus-formation produces a large leucocytosis which differentiates it completely from the other varieties.

In inflammations involving structures which cannot be well examined by the ordinary means at our command, such as the chamber of the middle ear or the interior of the skull, a white blood-count is of great value in that it tells us whether the process is catarrhal and no pus has formed, or whether the infection is pyæmic and has gone on to suppuration. In the one case no operative interference is needed ; in the other no delay should be tolerated. The writer well remembers a case of otitis media in which the clinicians were in doubt for some time as to whether the process was catarrhal or suppurative. A white count was made, showing 21,000 leucocytes to the c.mm., and the diagnosis of otitis media with pus-formation was made. Later the pus burrowed through the tympanum and discharged from the external ear.

Likewise, in the differential diagnosis between osteomyelitis and inflammations of bone of a tubercular or malignant variety a white blood-count assists in arriving at a correct conclusion, since in osteomyelitis there is almost always a leucocytosis, while in bone inflammations of the other varieties no leucocytosis occurs. Such knowledge would be of great value to the veterinarian who has to do with so many bone inflammations.

In these and many other conditions not here mentioned, but which will doubtless suggest themselves to you, the counting of the white blood-corpuscles of the blood is of practical clinical value to the veterinarian as well as to the physician.

Before proceeding to give in detail the white blood-counts in certain pus-formations, the writer wishes to call your attention to the method pursued. At first thought one might think the process too complicated for the ordinary practitioner, but I feel sure that such fears are not well grounded. The red blood mixer of a Thoma-Zeiss hæmocytometer and a bottle of $\frac{1}{3}$ per cent. glacial acetic-acid solution in water are constantly carried in a hand-satchel. When a patient is seen in which a white blood-count is desired to be made the ear is punctured with a needle, the mixer is filled with the proper amount of blood and acetic-acid solution. The apparatus is then well shaken, so as to thoroughly mix the blood and acid, the mixer placed in the case and taken to the office, where the count is made with the microscope. This method does away with the necessity for carrying an instrument to the place where the patient lives, and is accurate, providing the apparatus is well shaken previous to counting. The writer has found no loss of cells as a result of one or two hours' delay previous to making the count, and believes no error from loss of cells arises even though the estimation is not made at once. When accustomed to the procedure it should not require over fifteen minutes to make the count.

Bearing in mind that the normal blood-count is 7500 leucocytes to the c.mm., the counts made under the following conditions, where pus was present, and had been removed, will have their full significance: Swelling in groin, 12,600, 21,000; rectal abscess, 13,000; swelling in thigh, 13,300; appendicitis, 14,000, 16,000, 17,000, 19,200, 20,000; abscess in Douglas' cul-de-sac, 15,000; stitch abscess following laparotomy, 15,000; felon on finger, 17,000; otitis media, 17,600, 21,000, 25,600; pelvic abscess, 20,600, 31,000; boil on neck, 21,000; gum-boil, 27,000; breast abscess, 32,500; abscess in parotid gland, 35,000.

While the writer is not a veterinarian and has made few leucocyte-counts in septic infections of animals, his experience with counts made in human beings suffering from pus-formations in all parts of the body has been so generally of one result, namely, a well-marked leucocytosis where pus is found, and an absence of leucocytosis where pus is absent, that he unhesitatingly recommends this procedure as a means of determining whether the doubtful inflammatory processes of lower animals are of a purulent or non-purulent character.

